# The myth of the metabolic baseline: sleep–wake cycles undermine a foundational assumption in organismal biology

**DOI:** 10.1002/brv.70133

**Published:** 2026-01-22

**Authors:** Helena Norman, Daphne Cortese, Amelia Munson, Jan Lindström, Shaun S. Killen

**Affiliations:** ^1^ School of Biodiversity, One Health & Veterinary Medicine, College of Medical, Veterinary and Life Sciences University of Glasgow Glasgow G12 8QQ UK; ^2^ UMR Marbec Université Montpellier, Ifremer, INRAE, CNRS, IRD Sète France; ^3^ Department of Wildlife, Fish and Environmental Studies, Skogsmarksgränd Swedish University of Agricultural Sciences Umeå 901 83 Sweden

**Keywords:** metabolic rate, sleep variability, circadian energetics, energy allocation, intraspecific variation, bioenergetics, aerobic scope

## Abstract

Basal and standard metabolic rate (BMR and SMR) are cornerstones of physiological ecology and are assumed to be relatively fixed intrinsic properties of organisms that represent the minimum energy required to sustain life. However, this assumption is conceptually flawed. Many core maintenance processes underlying SMR are temporally partitioned across sleep and wakefulness and are not continuously active. We argue that instead of representing a singular metabolic state, SMR is better defined as a shifting metabolic mosaic where maintenance functions are distributed unevenly across different sleep–wake states, including metabolically and functionally distinct phases such as non‐rapid eye movement (NREM) and rapid eye movement (REM) sleep. SMR measured during wakefulness will mainly represent ion regulation, thermoregulation, sensory processing, and substrate cycling. Meanwhile, SMR measured during sleep primarily includes processes upregulated during sleep, including protein synthesis, cellular repair, immunity, and synaptic plasticity. Our models demonstrate that SMR values measured exclusively during wake or sleep consistently over‐ or underestimate daily maintenance costs depending on the time spent in specific sleep states and when SMR was measured. In addition, treatment or environmental effects on the costs of specific processes may be entirely missed if metabolic measures occur during an inappropriate sleep–wake state. The temporal partitioning of maintenance processes suggests that traditional and current approaches to SMR measurement may confound true metabolic variation with individual and species‐specific differences in sleep architecture, with implications for the estimation of energy budgets, trait heritability, environmental effects on metabolic rate, and metabolic scaling relationships. We propose redefining organismal maintenance costs as a time‐integrated profile of metabolic demands, but also suggest that state‐specific SMR measurements are appropriate if the sleep–wake measurement period aligns with that of the behavioural, physiological, or ecological context of interest. Moving beyond the fiction of a constant maintenance baseline would provide more refined insights into the bioenergetic foundations of ecological performance and evolutionary constraints.

## I. INTRODUCTION

Basal and standard metabolic rate (BMR and SMR) are among the most widely measured and applied traits in organismal biology. Both terms refer to the rate of energy throughput by a whole organism for baseline maintenance processes under standardised resting conditions. BMR applies to measurements at thermoneutral temperatures in endotherms, and SMR refers to measurements at any specified temperature in endotherms and ectotherms (Rolfe & Brown, 1997). The ubiquity of BMR and SMR (hereafter collectively referred to as SMR) in research stems from their scalability across biological levels, from cells to ecosystems, and their integration into foundational ecological theories (Kooijman, 1986, 2010; Brown *et al*., 2004). SMR often correlates with other fundamental organismal traits (e.g. growth rate; Burton *et al*., 2011), and is also used to derive traits such as aerobic scope (Halsey *et al*., 2018). These standardised estimates of energy use are fundamental across fields, influencing the study of life‐history strategies, responses to environmental change, species interactions, and population dynamics (White & Seymour, 2004; Norin & Metcalfe, 2019; Kozłowski, Konarzewski & Czarnoleski, 2020). Because SMR has been measured across an extraordinary diversity of taxa, it serves as a broadly comparable index of maintenance metabolism (Killen *et al*., 2016; Burger *et al*., 2021), even though absolute SMR values differ across species and the relative contribution of individual physiological processes to maintenance costs can vary among lineages (Pettersen, Marshall & White, 2018).

Standard metabolic measurements implicitly assume a constant baseline rate of energy use for maintenance processes in resting animals. In both concept and analytical practice, SMR is treated as an organism's stable minimal metabolic state, with short‐term fluctuations typically interpreted as ‘routine’ variation around this baseline and filtered out using standard extraction methods (Chabot, Steffensen & Farrell, 2016b). This conceptualisation assumes that cellular and physiological maintenance demands are continuously active and therefore measurable as a single, invariant SMR.

However, this approach masks a critical biological reality: different maintenance processes are upregulated or downregulated depending on what the animal is doing, with a particularly strong divide between wakefulness and sleep. Indeed, while some biological processes are downregulated during sleep (Berger & Philips, 1993), many others are upregulated (Schmidt, 2014). As a result, there is not a continuous metabolic baseline, but instead a metabolic mosaic of shifting maintenance energy demands that vary across the sleep–wake cycle based on which processes are active or suppressed. Indeed, a primary hypothesis for the function of sleep is an efficient energy reallocation among maintenance tasks that is incompatible with wakefulness (Schmidt *et al*., 2017). Consequently, SMR estimates taken during a single state – such as during sleep – capture only the maintenance costs that are predominant in that state, potentially misestimating both process‐specific and whole‐animal costs in other states.

Sleep is a remarkably conserved behavioural and physiological phenomenon, occurring across virtually all major branches of the animal tree of life, including insects, fishes, amphibians, reptiles, birds, and mammals, and even in taxa lacking centralised brains such as jellyfish and hydra (Stahel, Megirian and Nicol, 1984; Nath *et al*., 2017; Kelly *et al*., 2020; Rößler *et al*., 2022). Although the underlying mechanisms vary, most animals exhibit recurring periods of quiescence with reduced responsiveness, and sleep rebound after deprivation, that meet operational criteria for sleep (Campbell & Tobler, 1984; Norman *et al*., 2024). Importantly, sleep comprises multiple physiologically distinct states – such as non‐rapid eye movement (NREM) and rapid eye movement (REM) sleep in mammals and birds, or slow‐wave and active sleep in other vertebrates – each associated with different patterns of neural activity and tissue‐ or organ‐level metabolic demand. Some marine mammals and birds also exhibit unihemispheric NREM sleep (Mascetti, 2016), illustrating that sleep architecture is not only widespread but also evolutionarily flexible. This phylogenetic breadth highlights that state‐dependent partitioning of maintenance costs is relevant for any organisms that alternate between metabolically distinct quiescent and active states, as they will experience predictable shifts in baseline energy expenditure across the diel cycle. This is the case for virtually every animal species studied to date. In addition, because sleep states differ metabolically and vary in their duration across individuals and taxa, they introduce additional layers of temporal heterogeneity beyond the simple sleep–wake divide, meaning that the specific sleep states that occur during SMR measurement may substantially influence the resulting estimate of maintenance costs.

**Fig. 1:**
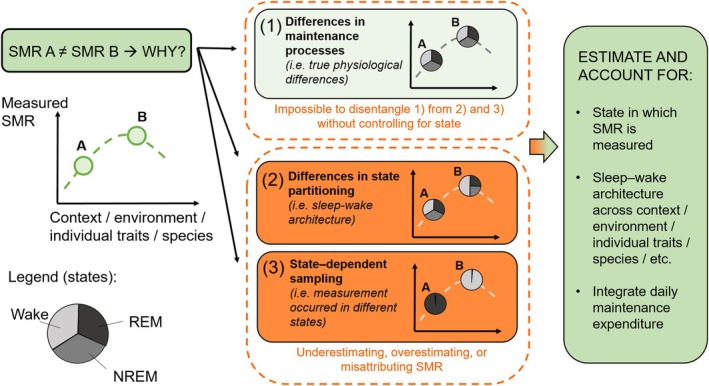
Disentangling physiological variation from state‐dependent effects and sampling artefacts. Differences in standard metabolic rate (SMR) between individuals, contexts, or species may reflect real variation in maintenance metabolism (1), but also differences in sleep–wake architecture (2) or the sleep–wake state during which measurements were conducted (3). Without controlling for sleep–wake state, these sources of variation are confounded, causing misestimation of whole‐animal SMR and treatment effects on specific maintenance processes. Pie charts illustrate proportions of time spent in wake, non‐rapid eye movement (NREM), and rapid eye movement (REM) states, which differ across contexts or individuals and affect SMR estimates. Accurate measurement and interpretation of SMR requires specifying the state in which measurements occur, quantifying sleep–wake architecture, or integrating across sleep–wake states to approximate daily maintenance expenditure.

Despite its ubiquity across animal taxa, sleep remains an underexplored source of variation in ecological and comparative physiology. Sleep architecture, which encompasses the duration, fragmentation, latency, and distribution of NREM and REM states, varies extensively among and within species. Across species, sleep duration and structure correlate with factors influencing SMR, including body size and life‐history characteristics (Savage & West, 2007). Within species, individuals show consistent differences in sleep architecture linked to behavioural types and energy budgeting (Randler, 2014). This variation may drive physiological differences often attributed to intrinsic metabolic traits. Specifically, SMR differences among treatments, individuals, or species, may reflect either: (*i*) true physiological variance in SMR; (*ii*) variation in sleep–wake architecture; or (*iii*) variation in the sleep–wake state during which SMR was measured – three potential sources of variation that are likely often conflated but must be disentangled for an accurate biological interpretation of SMR (Fig. 1). Environmental factors, such as temperature, photoperiod, and habitat structure, affect the time spent in different sleep–wake states (Rattenborg *et al*., 2008; Mortlock *et al*., 2024) and the corresponding maintenance costs, as could an animal's perception of the respirometry chamber, therefore further confounding metabolic measurements.

To utilise SMR fully as a meaningful physiological trait, we must recognise that it is not a fixed or static measure, but the sum of multiple maintenance processes that varies over time with sleep–wake state. Here, we synthesise literature on SMR's physiological components and their differential expression across sleep–wake states, broadly to estimate the state‐partitioning of maintenance functions. In doing so, we propose a fundamental shift towards redefining maintenance metabolism as a dynamic, state‐dependent profile shaped by sleep–wake cycles and suggest a re‐evaluation of how metabolic traits are measured, interpreted, and applied.

## II. WHY THERE IS NO STATIC SMR: THE CASE FOR STATE‐DEPENDENT PARTITIONING

**Fig. 2:**
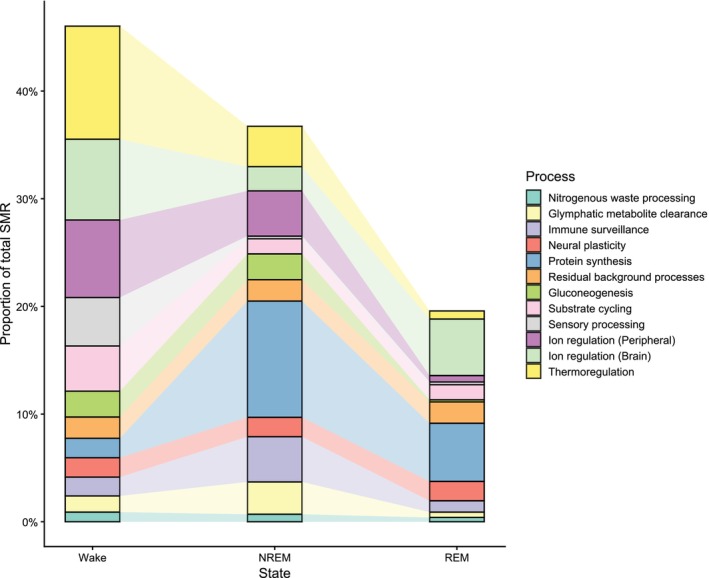
Estimated state‐dependent allocation of maintenance costs contributing to standard metabolic rate (SMR). Bars represent the proportion of total SMR attributable to different physiological maintenance processes when measured during wakefulness, non‐rapid eye movement (NREM) sleep, or rapid eye movement (REM) sleep, generated from the values in Table 1. Values are scaled such that the total SMR measured across all states sums to 100%.

To illustrate how state dependence can influence SMR estimation, we first break down SMR into its constituent maintenance processes, such as ion gradient maintenance, protein synthesis, and thermoregulation (see Fig. 2 and Table 1 for the full list of SMR maintenance processes). We then estimated the proportional contribution of each major maintenance process to overall SMR across sleep–wake states by using available estimates from the literature. However, for many processes, direct quantification of energetic costs across sleep–wake states does not exist. In these cases, we derived informed estimates by combining available data on the relative contribution of each process to total SMR with evidence for how the underlying physiological systems, organs, or cellular mechanisms are up‐ or down‐regulated across different sleep–wake states.

**Table 1:** Estimated state‐dependent partitioning of energy required for maintenance processes and the putative contribution of each process to standard metabolic rate (SMR). Depending on the process, state‐partition values were derived from a combination of direct physiological measurements and indirect evidence from gene expression, metabolic tracer studies, and neuroimaging. In most cases, contributions to SMR were estimated from published estimates, but these are typically based on data collected during a single behavioural state (e.g. sleep or quiet wakefulness); as a result, they may not reflect the full energetic cost of a function across the full sleep–wake cycle. Uncertainty rankings indicate the strength of empirical support (Low = well‐quantified; Moderate = indirect inference; High = speculative). We follow Rolfe & Brown (1997) in recognizing that organ‐level ‘service functions’ (e.g. liver detoxification, heart functioning, motor control of breathing) contribute significantly to whole‐body energy use. However, the energetic costs of these functions are already at least partially represented within the cellular processes included in our table (e.g. ion regulation, protein synthesis, substrate cycling), and their state‐dependent partitioning is difficult to resolve. For this reason, we do not treat service functions as a separate category.

Maintenance process	Partitioning (%)			Total SMR contribution (%)	State SMR contribution (%)			State uncertainty	SMR uncertainty
	Wake	NREM	REM		Wake SMR	NREM SMR	REM SMR		
Ion regulation (brain)	50	15	35	15	7.5	2.25	5.25	Moderate	Low
Ion regulation (peripheral)	60	35	5	12	7.2	4.2	0.6	Moderate	Moderate
Protein synthesis	10	60	30	18	1.8	10.8	5.4	Low	Low
Immune surveillance	25	60	15	7	1.75	4.2	1.05	Moderate	High
Thermoregulation	70	25	5	15	10.5	3.75	0.75	Low	Moderate
Neural plasticity	20	45	35	4	0.8	1.8	1.4	Low	Moderate
Sensory processing	90	5	5	5	4.5	0.25	0.25	Low	Moderate
Glymphatic metabolic clearance	30	60	10	5	1.5	3	0.5	Low	High
Nitrogenous waste processing	45	35	20	2	0.9	0.7	0.4	Moderate	Low
Substrate cycling	60	20	20	7	4.2	1.4	1.4	Moderate	Low
Gluconeogenesis	35	60	5	4	1.4	2.4	0.2	High	Low
Residual background processes	33	33	33	6	1.98	1.98	1.98	Moderate	Moderate
**TOTAL**				**100**	**44.03**	**36.73**	**19.18**		

NREM, non‐rapid eye movement; REM, rapid eye movement.

We recognise that this approach necessarily involves inference and that our quantitative estimates should be interpreted cautiously. However, we are not aiming to provide definitive quantitative estimates for all species and situations, but are instead demonstrating that maintenance processes are unlikely to be equally active across all sleep–wake states and offer a biologically informed heuristic for understanding how overlooking state partitioning can lead to systematic biases in SMR measurement. While our specific partitioning values lack precision, the broader biological reality that different maintenance functions are temporally partitioned across sleep and wakefulness is well established, and the metabolic consequences of this partitioning warrant additional quantitative research and consideration in metabolic rate studies.

In addition, the proportional allocations in Fig. 2 are drawn primarily from studies of humans and other mammals, for which studies are most abundant, and substantial variation likely exists across taxa, individuals, and environmental contexts. However, our broader logic of state‐dependent partitioning applies across taxa. The largest quantitative difference between endotherms and ectotherms concerns thermoregulatory costs: endotherms devote a substantial fraction of SMR to thermoregulation during wakefulness, whereas these costs are negligible in most ectotherms, potentially leading ectotherms to exhibit smaller absolute state‐dependent biases. Other sources of taxonomic variation are also expected, including brain ion‐regulatory costs that scale with both brain size and neuron density, and thus contribute differently to SMR across birds, mammals, reptiles, and fishes. For example, neurons in the avian pallium consume only about one‐third as much glucose as a typical mammalian cortical neuron (von Eugen *et al*., 2022), and brain metabolic rates differ widely among vertebrate groups (Heldstab *et al*., 2022). Nevertheless, although the *magnitude* of state‐dependent bias will vary across groups, the *direction* and qualitative form of the bias is likely to remain consistent. Because our aim is to illustrate the conceptual consequences of temporal partitioning rather than providing species‐specific numerical predictions, we do not model ectotherms separately here. However, we highlight that sleep–wake architecture is still expected to introduce systematic errors in ectotherms whenever maintenance processes differ across states, and defining these differences is a prime avenue for further research aimed at refining our estimates of maintenance costs in animals.

Below, we highlight the state‐dependent partitioning of several key maintenance processes that exemplify different patterns of temporal allocation across sleep–wake states.

### (1) Brain ion regulation

Maintaining ion gradients across neuronal membranes is one of the most energetically expensive functions in the brain. It has been estimated that ion pumping *via* Na^+^/K^+^‐ATPase accounts for roughly half of total cortical ATP consumption, particularly under awake conditions (Attwell & Laughlin, 2001; Howarth, Gleeson & Attwell, 2012). Given that the cerebral cortex accounts for approximately 20% of SMR in adult humans (Rolfe & Brown, 1997), this implies that cortical ion‐pumping alone accounts for ~10% of whole‐body SMR. However, this estimate excludes regions such as the thalamus, basal ganglia, cerebellum, and brainstem, which also maintain high baseline activity and demand for ion transport (Mokgokong *et al*., 2014; Hladky & Barrand, 2016). To account for these additional brain structures, we conservatively assign 15% of SMR to total brain ion‐gradient maintenance. During wake, cortical firing rates increase, driving maximal Na^+^/K^+^‐ATPase activity (Attwell & Laughlin, 2001; Howarth *et al*., 2012). During NREM sleep, neuron firing rates decline by ~40% (Vyazovskiy *et al*., 2008; Howarth *et al*., 2012), which should theoretically reduce Na^+^/K^+^‐ATPase activity proportionally. This is supported by metabolic evidence showing 25–44% reductions in brain glucose and oxygen metabolism during deep NREM sleep, with REM sleep showing partial rebound of firing rates (Dienel & Lauritzen, 2025). Based on this evidence, we partition overall brain ion regulation activity as 50% during wake, 15% during NREM sleep, and 35% during REM sleep (Fig. 2; Table 1).

### (2) Peripheral ion regulation

Even at rest, skeletal muscle consumes significant energy to maintain ionic balance. In resting human quadriceps, for example, Na^+^/K^+^‐ATPase and sarcoplasmic/endoplasmic reticulum Ca^2+^‐ATPase (SERCA) together account for approximately 25% of muscle oxygen use (Rolfe & Brown, 1997). Given that skeletal muscle comprises ~40% of human body mass, this implies a whole‐body contribution to SMR of ~10–12% from peripheral ion regulation. Although peripheral tissues like skeletal muscle and viscera require ionic gradient maintenance continuously, activity is also modulated by postural tone and other factors dependent on sleep–wake cycles, with high tonic muscle activation during waking periods necessitating increased Na^+^/K^+^‐ATPase and SERCA activity (Burgess *et al*., 2008). Conversely, muscle tone is reduced during NREM sleep and almost completely absent during REM sleep due to active brainstem inhibition of motoneurons (Siegel, 2005; Burgess *et al*., 2008; Vetrivelan & Bandaru, 2023). Therefore, we partitioned energetic demand for peripheral ion regulation as 60% to wake, 35% to NREM sleep and 5% to REM sleep (Fig. 2; Table 1).

### (3) Protein synthesis and cellular repair

Whole‐body protein turnover is a major component of maintenance metabolism, with protein synthesis and degradation together accounting for 18–25% of standard metabolic rate (Rolfe & Brown, 1997). Wakefulness is associated with basal protein turnover, but sleep – particularly NREM sleep – is the primary period for upregulation of genes involved in protein synthesis and folding (Mackiewicz *et al*., 2007; Puentes‐Mestril *et al*., 2021). Additional support comes from sleep‐deprivation studies showing that prolonged wake suppresses these pathways, which rebound during recovery sleep (Naidoo, 2009). Accordingly, we have partitioned these costs to reflect higher biosynthetic activity during sleep (Fig. 2; Table 1).

### (4) Thermoregulation

Thermoregulation is a key component of maintenance metabolism in endotherms, typically accounting for approximately 10–15% of SMR under resting conditions at or near thermoneutrality (Rolfe & Brown, 1997). While this cost primarily reflects active thermoregulatory control mechanisms, sleep strongly modulates these thermoregulatory activities in a state‐dependent manner. During wakefulness, thermoregulatory reflexes are fully functional, allowing precise control of core body temperature (Tan & Knight, 2018). NREM sleep involves a downward shift in the thermoregulatory setpoint rather than a loss of thermoregulatory control. Although animals can still activate thermoeffectors such as panting or shivering, NREM sleep is nevertheless characterised by reductions in core and cortical temperature and by decreased responsiveness to thermal challenges (Tan & Knight, 2018; Harding, Franks & Wisden, 2020). Although heat continues to be produced by basal metabolic processes, the defensive mechanisms that maintain temperature set points are downregulated. During REM sleep, thermoregulatory defences are almost entirely disengaged or suppressed (Schmidt, 2014; Harding *et al*., 2020), possibly to reallocate resources to neural processes (Cerri *et al*., 2017). Based on this evidence, we allocate 70% of thermoregulatory energy expenditure to wakefulness, 25% to NREM, and 5% to REM.

### (5) Neural plasticity/memory consolidation

Neural plasticity refers to the restructuring of synaptic connections through strengthening, weakening, or remodelling, and involves energy‐intensive activities such as protein synthesis, receptor modulation, and neural reorganisation. Based on *in vivo* ATP imaging during NREM sleep, it is estimated that ~10% of cortical ATP during sleep is directed towards plasticity‐related processes (Seibt *et al*., 2012). When scaled to the cortex's overall contribution to total SMR (~20% in humans; Rolfe & Brown, 1997) and expanded to account for additional plasticity demands in subcortical and glial structures, we estimate that neural plasticity contributes approximately 4% of whole‐body SMR. Plasticity is not distributed evenly across behavioural states; as described by the synaptic homeostasis hypothesis (Tononi & Cirelli, 2014), synaptic strength accumulates during wakefulness as new information is encoded, but is selectively downscaled during NREM sleep to restore efficiency. These NREM‐linked changes are supported by dendritic calcium bursts that coincide with sleep spindles and signal localised increases in energy use (Seibt *et al*., 2017). REM sleep contributes a second wave of plasticity, characterised by hippocampal–cortical replay of activity patterns from prior waking experience, thought to underlie memory consolidation (Levenstein, Buzsáki & Rinzel, 2019). Reflecting the combined but distinct contributions of NREM and REM, we allocate 45% of plasticity‐related energy use to NREM, 35% to REM, and 20% to wake.

### (6) Sensory processing

Sensory processing is a metabolically active function of the cortex, particularly during wakefulness when animals must monitor and respond to external stimuli. Sensory cortical regions (e.g. visual, auditory, and somatosensory cortices) represent a major share of cortical volume and synaptic activity during wakefulness. Given that the cerebral cortex accounts for ~20% of whole‐body SMR (Rolfe & Brown, 1997), and that a significant portion of cortical signalling is dedicated to sensory integration during wake, it is reasonable to estimate that sensory processing contributes ~3–5% of SMR. This does not include energy used by subcortical sensory relays (e.g. thalamus) or alertness‐related sensory gating. To account conservatively for these components and reflect continuous sensory engagement during wake, we assign 5% of SMR to sensory processing. Sensory cortical activity declines significantly during NREM sleep (Tagliazucchi & Laufs, 2014) and is largely disengaged during REM sleep despite high overall brain activity (Siegel, 2005).

### (7) Immune surveillance and modulation

Estimates of the energy cost of baseline immune function are not available, but both theoretical and empirical data suggest that constitutive immune processes (e.g. leukocyte maintenance, low‐level cytokine signalling, and general immune readiness and surveillance) represent a non‐trivial portion of the resting metabolic rate (Lochmiller & Deerenberg, 2000; Demas, 2004). Most empirical work focuses on activated immune responses (which can raise metabolism by 15–30%), while the baseline maintenance of immune competence likely involves continuous low‐level metabolic investment from lymphoid organs and leucocyte activity, suggesting these costs are present even in healthy individuals (Aho *et al*., 2013). Based on this, a conservative estimate of ~7–8% of SMR for baseline immune metabolism is biologically plausible, although uncertainty is high due to the lack of direct quantification. Evidence suggests that baseline immune surveillance is not uniformly distributed across the sleep–wake cycle, but instead exhibits functional partitioning shaped by circadian and behavioural state (Besedovsky, Lange & Born, 2012; Shivshankar *et al*., 2020). Indeed, circadian activity of some immune components (e.g. cytokines) appears to have neuromodulatory roles that regulate sleep, in addition to their direct immunological function (Motivala & Irwin, 2007). Studies in humans and animals also show that early NREM sleep coincides with a hormonal response that favours immune coordination, characterised by low cortisol and high growth hormone levels, with NREM sleep supporting adaptive immune functions such as antigen presentation, leukocyte activity, and T‐cell activity (Besedovsky, Lange & Haack, 2019). Disruptions to sleep reliably alter immune gene expression (e.g. Aho *et al*., 2013), further suggesting that normal sleep facilitates important immune processes. By contrast, REM sleep is thought to contribute little to baseline immune functioning, as it coincides with rising cortisol levels, reduced growth hormone levels, and increased sympathetic activation (Besedovsky *et al*., 2019). Together, these findings support a model in which NREM sleep is the primary period of baseline immunological maintenance and coordination, while wake supports more peripheral immune readiness and REM sleep contributes minimally (Table 1). However, the precise energetic costs of these processes remain uncertain and likely vary across species, tissues, and immune functions.

### (8) Glymphatic metabolite clearance

Glymphatic clearance is the convective exchange of cerebrospinal and interstitial fluid that facilitates metabolic waste removal from the brain, and is upregulated during sleep. While the energetic cost of this process has not been directly quantified, it likely imposes appreciable ATP usage associated with glial activity, cerebrospinal fluid movement, and vascular–neural coupling. Based on this rationale, we provisionally estimate glymphatic function to contribute approximately 5% of SMR, reflecting the likely contribution of glial and vascular processes during peak glymphatic activity, but this estimate should be interpreted cautiously due to the absence of direct measurements. Glymphatic function is strongly state dependent. During NREM sleep, there is a twofold increase in clearance compared to wake (Xie *et al*., 2013), then a reduction during REM sleep (Rasmussen, Mestre & Nedergaard, 2018). Based on this evidence, we assign 60% of glymphatic metabolic activity to NREM sleep, 30% to wake, and 10% to REM sleep.

### (9) Nitrogenous waste processing

It has been estimated that nitrogenous waste management contributes approximately 2% of SMR in mammals (Rolfe & Brown, 1997). The temporal expression of nitrogenous waste processing exhibits pronounced circadian partitioning linked to both protein turnover cycles and kidney function rhythms, although the evidence for changes in metabolic costs remains mostly indirect and inferred through changes in kidney activity. Glomerular filtration rates, for example, display strong circadian rhythmicity, with maximum values during daytime and minimum values at night (Knutson *et al*., 2018). Experimental evidence demonstrates that renal hormonal control differs fundamentally between sleep and wake states (Charloux *et al*., 1999). During sleep, aldosterone pulses are mainly related to plasma renin activity (PRA) oscillations, whereas during waking periods, aldosterone pulses are primarily associated with cortisol pulses. Furthermore, PRA shows oscillations strongly linked to REM–NREM cycles, with NREM sleep linked to increasing PRA and REM sleep associated with decreased PRA (Charloux *et al*., 1999). These state‐dependent differences in renal regulation suggest that metabolic costs of nitrogenous waste processing vary across states. Based on these considerations and documented circadian variations in hepatic and renal function, we provisionally assign 45% of nitrogenous waste processing costs to wakefulness, 35% to NREM sleep, and 20% to REM sleep, although uncertainty surrounds these estimates given limited direct quantification of state‐dependent waste metabolism across different taxa.

### (10) Substrate cycling

Substrate cycling refers to ATP‐consuming biochemical loops involving opposing metabolic pathways (e.g. glycolysis and gluconeogenesis, lipolysis and lipogenesis, triglyceride and fatty acid turnover) that allow rapid shifts in fuel usage and metabolic regulation. Rolfe & Brown (1997) estimated that substrate cycling contributes approximately 7.5% of SMR, based on modelling of futile cycles across liver, muscle, and adipose tissue. More recent studies (e.g. Zhang *et al*., 2021) have shown that substrate switching – indicated by fluctuations in respiratory quotient – continues across the sleep–wake cycle, particularly during transitions in and out of REM sleep, supporting the view that although substrate cycling is most pronounced during waking periods, when metabolic rate, sympathetic tone, and fuel demands are highest, it is also modulated by sleep. NREM sleep, despite its overall energy‐conserving profile, is associated with increased lipolysis, growth hormone release, and free fatty acid availability, all of which support ongoing hepatic and adipose substrate cycling (Copinschi, Leproult & Spiegel, 2014; Adamantidis, Gutierrez Herrera & Gent, 2019). During REM sleep, bursts of sympathetic activity and increased brain glucose uptake further sustain metabolic flexibility. Based on this, we assign 60% of substrate cycling energy use to wakefulness, and 20% each to NREM and REM sleep, reflecting both continuous background cycling and state‐specific modulation.

### (11) Gluconeogenesis

Gluconeogenesis and glycogen metabolism are essential components of energy homeostasis, particularly during fasting or extended periods without food intake, such as overnight sleep. Although their energetic cost is often overlooked, Rolfe & Brown (1997) estimated that gluconeogenesis could account for 3–6% of SMR in mammals. This includes both hepatic glucose production and brain glycogen turnover, which is known to fluctuate across behavioural states. According to the glycogenetic hypothesis, brain glycogen is depleted during wakefulness due to high neuromodulatory activity and replenished during NREM sleep, a process supported by increased glycogen synthase activity and reduced sympathetic tone (Petit *et al*., 2015). By contrast, glycogen breakdown and gluconeogenic demand rise during wakefulness, particularly during prolonged wake or energy stress, when glucose needs increase in both peripheral tissues and the brain. REM sleep appears to contribute minimally to net glycogen turnover, although bursts of neuronal activity may elevate local glucose oxidation. Based on these patterns, we estimate the contribution of gluconeogenesis and glycogen metabolism to SMR at 4%, and partition the cost as 60% to NREM sleep, 35% to wakefulness, and 5% to REM sleep, reflecting the anabolic and catabolic phases of carbohydrate cycling across the sleep–wake cycle.

### (12) Residual background processes

A portion of standard metabolic rate reflects baseline cellular functions that are essential for survival but not strongly influenced by behavioural state. Rolfe & Brown (1997) estimated that these background processes account for approximately 5–8% of SMR, depending on the species and tissue type. This component is thought to remain relatively constant across sleep–wake states, as it reflects the irreducible energetic cost of maintaining basic membrane potential, resting mitochondrial function, and low‐level enzymatic activity. For this reason, we assign a representative value of 6% of SMR to background maintenance functions and apply it evenly across wake, NREM sleep and REM sleep.

### (13) Overall analysis

These trends suggest that there is no singular or fixed value for SMR and that organismal maintenance costs are therefore best understood as an integrated outcome of shifting processes that are differentially expressed across sleep–wake states. Although wakefulness often involves upregulation of energetically intensive functions such as sensory processing, ion‐gradient maintenance, and thermoregulation, sleep is not metabolically uniform. Different sleep states – such as light and deep NREM or REM sleep – exhibit distinct patterns of neural, somatic, and thermoregulatory activity, and likely differ in the costs associated with specific maintenance processes. Indeed, additional variation almost certainly exists within these states (e.g. variation in NREM depth or REM phasic activity), but our heuristic model treats each state as a single category to illustrate the broader principle of state‐dependent partitioning. As a result, any SMR measurement taken during one state is likely to capture only a subset of total maintenance costs. Crucially, the extent and direction of this bias depends on the organism's sleep architecture, which varies among individuals, contexts, and species.

An important caveat to our analysis is that the baseline SMR values and process contributions underlying Table 1 have been derived from studies that were likely affected by the same sleep–wake biases we discuss here, being measured under uncontrolled or unspecified sleep–wake conditions. As such, they may already reflect state‐dependent sampling artefacts instead of true physiological costs. This creates a somewhat circular problem, because we are using potentially biased data to quantify the magnitude of bias in metabolic rate measurements. However, this limitation highlights a key motivation for measurement refinements and the conceptual shift we are proposing. Not only are new methods needed to improve future studies, but they are required to validate (or correct) existing metabolic rate data retrospectively that may have been affected by unrecognised sleep–wake artefacts.

It is also worth noting that, where differences in SMR among taxa or treatments span orders of magnitude (e.g. mouse–elephant comparisons), sleep‐state‐related bias may have little influence on broad qualitative comparisons between species or treatments, because the distributions will not overlap regardless of measurement state. Nevertheless, even when smaller than the among‐group contrast, unaccounted variation in sleep–wake state will still inflate residual variance and can bias estimates of effect sizes, scaling exponents, or heritability, particularly when comparing individuals, treatments, populations or closely related species with overlapping metabolic ranges.

## III. CONSEQUENCES OF OVERLOOKING STATE‐DEPENDENT MAINTENANCE COSTS

**Fig. 3:**
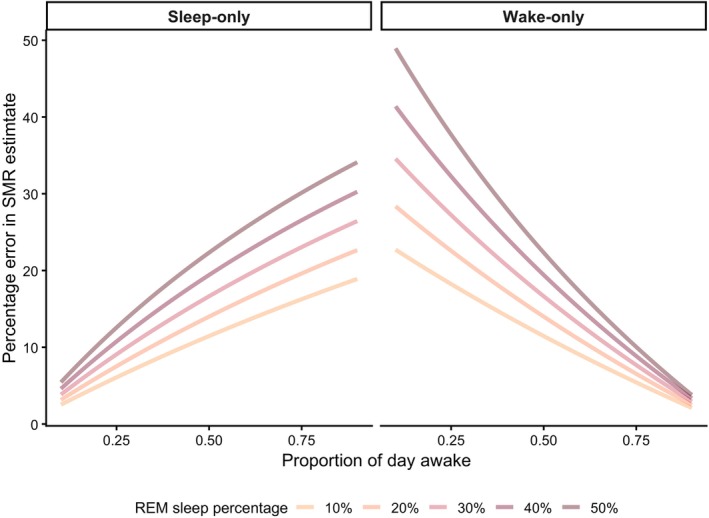
Predicted error in standard metabolic rate estimation when measured exclusively during wake or sleep. Per cent error in estimated standard metabolic rate (SMR) is shown as a function of the proportion of the day normally spent awake for a given individual or species, assuming that SMR is measured only during sleep (left) or only during wakefulness (right). Errors are expressed relative to the true integrated 24‐h SMR. The model assumes a fixed contribution of each state to SMR [wake = 44%, non‐rapid eye movement (NREM) sleep = 37%, rapid eye movement (REM) sleep = 19%; Fig. 2; Table 1]. For illustrative purposes, we restricted the model to individuals that spend between 10 and 90% of the day awake, since animals that never sleep or never wake are biologically unrealistic and cannot, by definition, be measured in the ‘sleep‐only’ or ‘wake‐only’ scenarios.

**Fig. 4:**
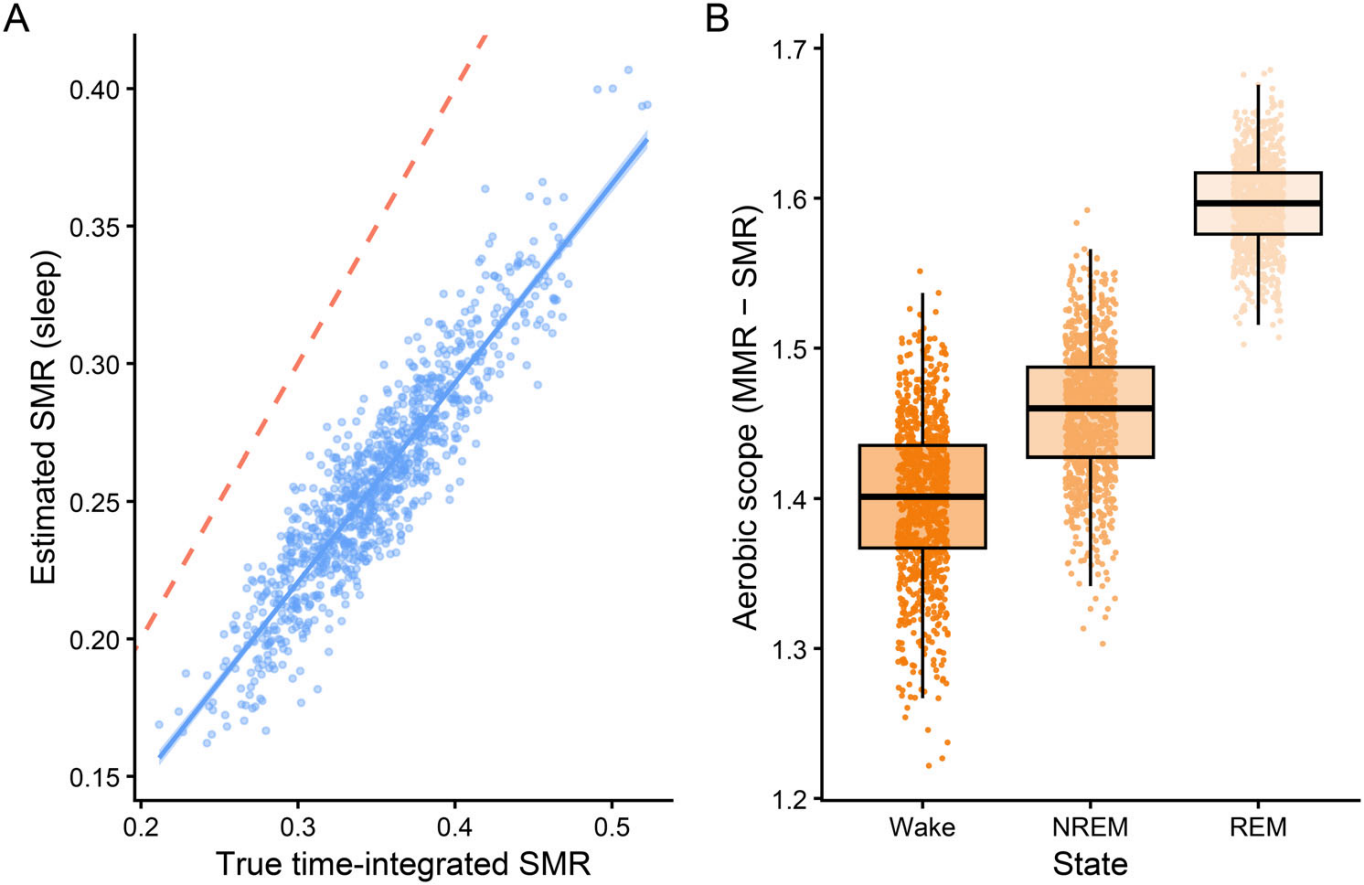
Simulated effects of overnight measurements on estimates of standard metabolic rate (SMR) and aerobic scope, and the influence of sleep architecture. (A) Each point represents a simulated diurnal individual (*N* = 200), measured on each of five nights (five points per individual). The *x*‐axis shows true standard metabolic rate (SMR), defined as the ideally measured and time‐integrated average over 24 h and accounting for partitioned energy use across wake, non‐rapid eye movement (NREM), and rapid eye movement (REM) states. The *y*‐axis shows the SMR that would be estimated if measured during a fixed 12‐h overnight window. The dashed red line represents points where sleep‐estimated SMR equals the true time‐integrated SMR. (B) Aerobic scope [maximum metabolic rate (MMR) – SMR] is shown for each sleep–wake state, based on simulated state‐specific SMR values. Metabolic rates are expressed in arbitrary units scaled to the simulated population distribution (true SMR mean = 0.35; see Appendix S1).

**Fig. 5:**
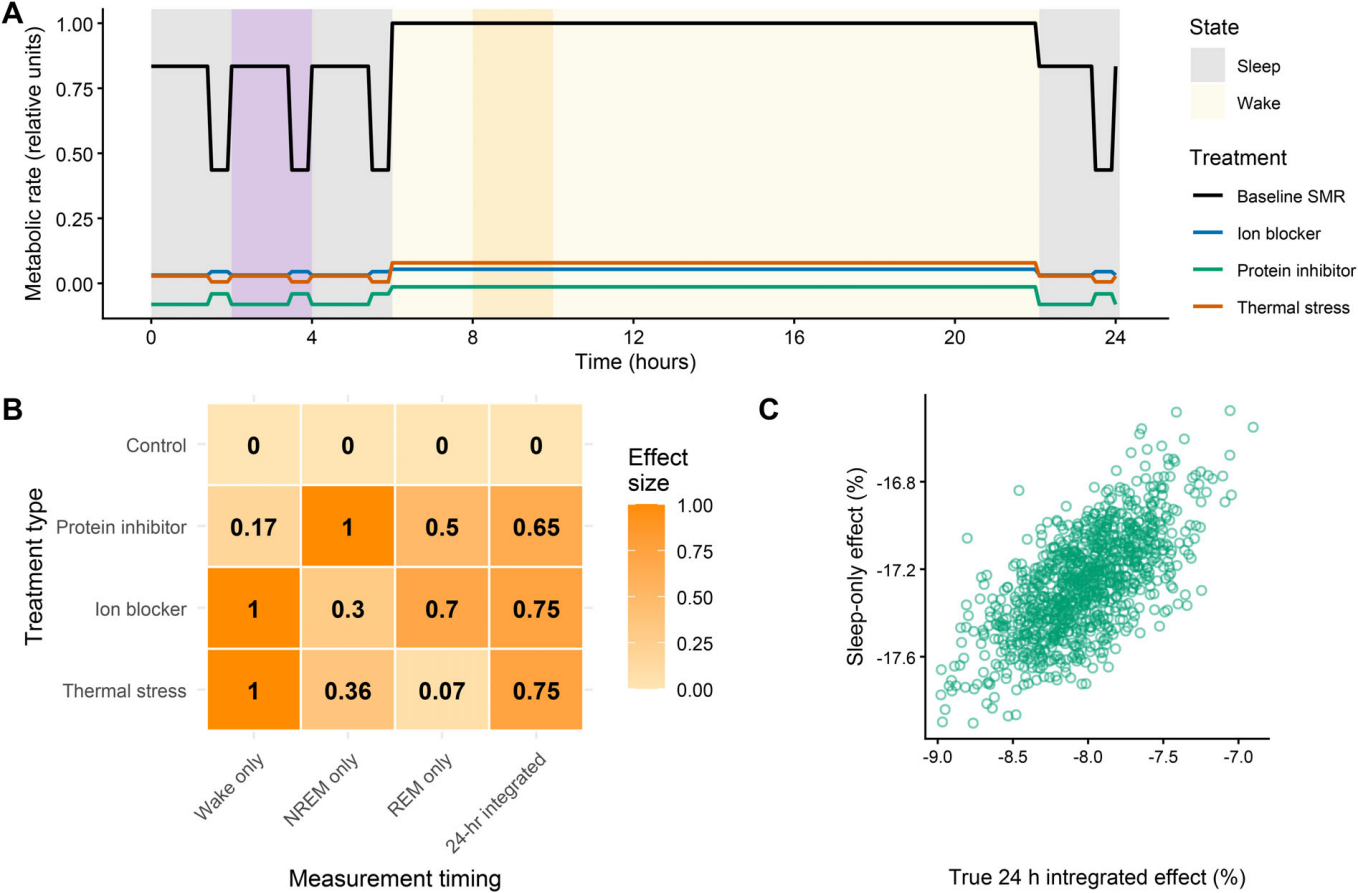
Consequences of state‐dependent maintenance costs for detecting treatment or environmental effects on metabolic rate. (A) Simulated 24‐h timeline of standard metabolic rate (SMR) under baseline and treatment conditions. The black line represents the shifting SMR baseline, calculated from the integrated costs of all maintenance processes, which vary with behavioural state [wake, non‐rapid eye movement (NREM) sleep, rapid eye movement (REM) sleep; Fig. 2]. Coloured lines show treatment‐induced metabolic costs for three hypothetical experimental manipulations: a protein synthesis inhibitor (green), an ion transport blocker (blue), and thermal stress (orange). Costs for each process are relative to baseline SMR at any timepoint, and relative to the background cost of each process (0 = no change from process background). Shading denotes periods of wakefulness (light yellow) and sleep (grey); purple represents a period of sleep metabolic rate measurement; dark yellow represents a period of wake measurement. (B) Heat map showing how treatment effects depend on the timing of SMR measurements. Values indicate the proportion of maximum treatment effect that would be detected if sampling were restricted to wake, NREM sleep, REM sleep, or a 24‐h integrated period. Each treatment shows maximum effect size (1.0) during the sleep–wake state when the targeted maintenance process is most active: protein synthesis during NREM sleep, ion regulation during wake, and thermoregulation during wake. (C) Results of simulation showing the discrepancy between estimated treatment effects of a protein‐synthesis inhibitor, based on sleep‐only sampling and the true 24‐h integrated effect, across 200 individuals over 5 days each (see Appendix S1). Each point represents a single individual‐day. Treatment effects of the hypothetical protein inhibitor were applied only to sleep‐active processes, and individual variation in sleep duration and REM:NREM ratio causes systematic bias when sampling is restricted to sleep, relative to the total time‐integrated SMR. Both axes show treatment effects as per cent change relative to the total time‐integrated value for SMR.

The unequal partitioning of maintenance processes across sleep and wakefulness creates two broad problems: (*i*) sleep architecture will influence whole‐animal SMR estimation and misrepresent maintenance costs (Figs 3 and 4); and (*ii*) measuring SMR during only one state accounts for only a portion of maintenance processes, and so the magnitude of observed effects of a factor or treatment on SMR will vary depending on the maintenance processes that are affected and the state in which SMR is measured (Fig. 5). For example, if a wake‐predominant process like substrate cycling is upregulated due to a treatment, sleep‐only SMR measurements will not accurately reflect this change in maintenance costs.

### (1) Estimating error in SMR from state‐limited sampling

To examine the first type of error, we developed a model that explored how state‐specific SMR measurements diverge from true 24‐h maintenance costs under varying sleep–wake schedules and REM sleep proportions (Fig. 3; see online supporting information Appendix S1 for details). This simulation illustrates the error produced when SMR is estimated solely during either sleep or wakefulness, using partitioning estimates shown in Fig. 2 and Table 1. When SMR is measured exclusively during sleep (Fig. 3A), estimated values increasingly underestimate the true integrated 24‐h maintenance costs as the proportion of the day normally spent awake increases. On the other hand, if SMR is measured only during wakefulness (Fig. 3B), estimates increasingly overestimate true 24‐h maintenance costs as the duration of unmeasured sleep increases. Notably, both types of bias will be exacerbated in individuals or species with a higher proportion of REM sleep, which is associated with particularly low metabolic activity.

### (2) Effects of state‐dependent partitioning on SMR and aerobic scope estimation

We then developed an individual‐based model to examine how variation in sleep architecture may bias estimates of both SMR and aerobic scope (see Appendix S1 for details). Absolute aerobic scope is the difference between an organism's maximum metabolic rate (MMR) and its SMR, and represents the metabolic capacity available for simultaneous aerobic processes above maintenance costs, including locomotion, digestion, and behavioural activity (Fu, Dong & Killen, 2022). Specifically, we simulated repeated overnight measurements for a population of individuals differing in their true integrated SMR, total sleep duration, and proportion of REM sleep, allowing us to examine how these factors interact to produce misestimates of SMR. We found that although the proportion of SMR missed during sleep‐only sampling remains constant, individuals with higher true SMRs experience larger absolute errors (Fig. 3A). Importantly, this error in SMR estimation carries over to affect calculations of aerobic scope (Fig. 3B). Although MMR is generally measured during wakefulness and little is known about whether it varies across the circadian cycle, some underlying physiological determinants of maximal performance (e.g. mitochondrial efficiency, endocrine status, body temperature) can exhibit circadian modulation. This raises the possibility, which is currently untested in most species, that MMR itself could vary across the diel cycle. In our model, we therefore held MMR constant for simplicity, but SMR varied due to differing maintenance demands during wakefulness, NREM, and REM sleep. Consequently, using sleep‐based SMR measurements to infer aerobic capacity may overestimate the performance capacity achievable during wakefulness. Notably, calculations of aerobic scope provide an example where state‐specific SMR values, rather than a 24‐h integrated SMR value, are most appropriate, since the latter would reflect average aerobic capacity across a circadian cycle, whereas state‐specific values more accurately represent performance limits relevant to the behavioural or ecological context being studied during a given state (e.g. wakefulness).

### (3) Impact of state‐restricted SMR measurements on detection of treatment effects

To examine how state‐dependent maintenance processes can bias estimates of treatment or environmental effects on SMR estimates, we developed an individual‐based model that simulates how SMR and specific process costs are partitioned across sleep–wake states (see Appendix S1 for model details). A hypothetical inhibitor treatment was applied in this simulation that reduced costs of protein synthesis by 50% across all sleep–wake states. Treatment effects were calculated both from the 24‐h integrated SMR estimates, and those based only on measurements during sleep (NREM and REM), while allowing individuals to vary in total sleep duration and the proportion of REM within sleep. Due to the upregulation of protein synthesis during sleep (Mackiewicz *et al*., 2007), sleep‐only measurements show a greater effect size as compared to the true integrated daily treatment effect (Fig. 4A, C), suggesting that apparent among‐individual variability in treatment responses may partly reflect differences in sleep architecture, rather than true physiological heterogeneity. For example, a treatment that alters thermoregulatory costs or ion regulation (predominantly active during wakefulness), or protein synthesis (mainly during sleep), could be substantially underestimated or entirely missed if measurements are taken during the wrong state (Fig. 4A, B).

## IV. ECOLOGICAL AND EVOLUTIONARY CONSEQUENCES

### (1) Sleep–wake partitioning as an evolutionary constraint

The partitioning of maintenance functions across sleep–wake states has implications for evolutionary constraints on maintenance metabolism. If specific maintenance processes are prioritised during specific sleep stages, then their expression is limited by the amount and architecture of sleep that an organism can accommodate. For example, enhanced cellular repair or immune function may require increased NREM sleep, but this may be evolutionarily constrained in species facing high predation risk or strong selection for vigilance (Lesku *et al*., 2008). Conversely, species with consolidated or prolonged sleep may afford investment in metabolically costly processes that occur during sleep, facilitating different metabolic adaptations. Overall, selection on maintenance efficiency could favour specific sleep patterns, while ecological sleep constraints may limit evolutionary options for maintenance costs. These linkages could generate relationships among sleep architecture, behaviour, and physiology that may remain undetectable if maintenance costs are measured without accounting for sleep–wake state, masking potential roles for sleep architecture as a hidden axis of life‐history trade‐offs and physiological trait evolution.

### (2) Repeatability and heritability of metabolic traits

Repeatability describes trait consistency within individuals, while heritability estimates the proportion of trait variation that is due to genetic differences. Both measures determine whether traits like SMR are likely to respond to selection. However, if metabolic measurements are influenced by unmeasured variation in sleep architecture, SMR estimates may reflect transient sleep–wake states, as opposed to stable physiological traits. This would artificially inflate within‐individual variability and reduce repeatability, especially if sleep patterns fluctuate across measurement days. Conversely, if sleep architecture is stable within individuals but varies consistently among individuals, the corresponding sleep‐biased SMR estimates may appear more repeatable than the true 24‐h integrated maintenance expenditure (Steinmeyer, Kempenaers & Mueller, 2012; Alós, Martorell‐Barceló & Campos‐Candela, 2017) and SMR heritability estimates may partially reflect genetic variation in sleep architecture rather than maintenance metabolism.

### (3) The scaling of metabolic rates with body mass

For more than a century, researchers have sought general ‘scaling laws’ describing how metabolic rate changes with body size (Kleiber, 1947; McNab, 1997; White & Seymour, 2003; da Silva, Garcia & Barbosa, 2006). However, accumulating evidence suggests that metabolic scaling exponents vary systematically with species lifestyle (Killen, Atkinson & Glazier, 2010; Carey, Sigwart & Richards, 2013), thermal environment (Kwak, Im & Shim, 2016; Ong *et al*., 2017), ontogenetic stage (Glazier, 2006), and activity level (Carey *et al*., 2013; Glazier & Gjoni, 2024). Our framework suggests that part of this variation may stem from overlooked differences in sleep–wake architecture across body sizes. Traditional scaling models assume that SMR reflects consistent maintenance processes across organisms, but if these processes are differentially expressed across sleep–wake states, and sleep‐state partitioning varies with body size, SMR scaling may include hidden biases. Smaller animals sleep more but have shorter, fragmented cycles and higher relative SMRs (Savage & West, 2007), meaning that over a given time interval, their SMR measurements may sample a broader range of sleep–wake states. Larger animals tend to exhibit reduced but more consolidated sleep (Savage & West, 2007), potentially producing more state‐specific but less representative SMR measurements. Furthermore, if specific maintenance functions scale differently with body mass and are differentially regulated across sleep states, apparent scaling exponents may reflect sampling‐window bias or process‐specific measurement bias, rather than fundamental physiological rules, introducing unrecognised variability into metabolic scaling.

### (4) Ontogenetic patterns and developmental energetics

Sleep architecture changes markedly during early development (Lesku, Martinez & Rattenborg, 2009). Altricial mammals can spend 80–100% of their early postnatal sleep in REM, whereas precocial species maintain relatively stable, adult‐like sleep‐state proportions from birth or hatching (Jouvet‐Mounier, Astic & Lacote, 1969). Because REM sleep is associated with particularly low metabolic activity, SMR measured during sleep in young altricial animals may underestimate true maintenance costs, potentially confounding species comparisons of developmental energetics or intraspecific metabolic scaling. Moreover, because costly processes such as protein synthesis and neural plasticity are preferentially active during sleep, SMR estimates incorporating wake periods may underestimate the energetic costs of growth and brain development in young animals. While sleep architecture continues to change across entire lifespans, exact patterns differ among species. In humans, for example, sleep quality and consolidation decreases with aging, while laboratory rodent studies show increasing sleep duration and intensity with age (McKillop & Vyazovskiy, 2020; Campos‐Beltrán & Marshall, 2021). Overall, age‐related changes in sleep may create measurement biases that vary both across species and throughout lifespans, with potential implications for comparative studies of aging and senescence.

### (5) Links between metabolic traits and behaviour

Over the last two decades, interest has surged in quantifying relationships between SMR and behaviour (Mathot, Dingemanse & Nakagawa, 2019; Bailey *et al*., 2022). However, if maintenance costs differ systematically between sleep and wakefulness, then measuring SMR during sleep and behavioural data during wakefulness is effectively sampling from different physiological baselines. As such, using SMR values from one state to predict behaviour may be meaningless without cross‐state correlation in total maintenance costs between wakefulness and sleep. This issue may also obscure observations of behavioural syndromes. Differences in boldness, vigilance, or exploration could influence how individuals sleep within respirometry apparatus, with more timid individuals sleeping less deeply or more briefly. These differences would affect measured SMR, creating spurious correlations between metabolism and wake‐measured behavioural phenotypes driven by sleep–wake state variation during SMR measurement. In addition, SMR–behaviour correlations commonly vary across environmental gradients like temperature or food availability (Killen *et al*., 2013), and this phenomenon is typically interpreted as an environmentally induced shift in trait covariance. However, some observed covariance shifts may reflect artefacts from inconsistent sleep state partitioning during SMR estimation, especially if environmental factors alter sleep architecture. Additionally, single‐state SMR measurements only capture a subset of specific maintenance processes, so correlation variations may be due to shifts among sleep‐predominant maintenance processes, while relationships involving waking processes may remain unobserved and therefore undetectable.

Recent work also shows that social stress and dominance hierarchies can alter individual sleep architecture, with dominant and subordinate individuals differing in REM duration and sleep fragmentation (Karamihalev *et al*., 2019). Aside from direct effects of sleep variation on metabolic costs and recovery from conflict, the widely observed associations between SMR and aggression or dominance (Milewski *et al*., 2022) may be partly mediated by variable sleep–wake states during SMR measurement between dominants and subordinates, and not solely due to intrinsic metabolic phenotypes.

### (6) Thermal performance curves of aerobic scope

Thermal performance curves for aerobic scope are widely used to assess physiological limits of ectotherms under different thermal environments, identify thermal optima, and predict climate change vulnerability (Khelifa *et al*., 2019). However, sleep–wake partitioning of SMR could introduce unacknowledged error in aerobic scope estimation across temperatures. Since SMR is often measured during resting periods that may include varying sleep proportions, temperature‐driven shifts in sleep duration or architecture may systematically bias SMR estimates. Additionally, if animals sleep more deeply or longer at certain temperatures, and if these sleep states involve lower metabolic costs, SMR measured during those periods will be artificially low. This would inflate aerobic scope estimates due to reduced maintenance costs captured during sleep‐heavy measurement windows, as opposed to true physiological optimisation. Such biases could affect thermal performance curves by exaggerating peaks, shifting optima, or confounding performance limits, all due to effects of temperature on sleep architecture in addition to direct effects on SMR itself. Moreover, interspecific or interindividual comparisons could become complex if temperature sensitivity of sleep differs among taxa or individuals, as these differences could appear as variation in aerobic performance instead of measurement artefacts.

### (7) Calming effects of conspecifics and social buffering of stress

Numerous studies report that the presence of conspecifics reduces measured metabolic rates in social species, often interpreted as a calming or stress‐buffering effect (Kikusui, Winslow & Mori, 2006; Gilmour & Bard, 2022). However, if these measurements are taken during quiescent periods (e.g. during the night in diurnal animals), an alternative explanation is that conspecific presence modulates sleep architecture (Beauchamp, 2009), leading to changes in the proportions of REM and NREM sleep being observed. For example, decreased risk perception in the presence of conspecifics may lead to longer or deeper NREM sleep (Tisdale *et al*., 2018), or less‐fragmented sleep cycles, thereby reducing the contribution of metabolically costly waking states during the SMR measurement window. Conversely, isolation or social stress might fragment sleep or increase the time spent awake, elevating apparent SMR. This would mean that the observed metabolic changes may reflect indirect shifts in the sleep‐state composition during measurement instead of direct decreases in maintenance or routine costs *via* stress reduction. If true, this reinterpretation could alter how we view social buffering effects and their implications for energy budgets in group‐living species.

### (8) Improving our understanding of environmental change

Our framework suggests that environmental change research faces two challenges: (*i*) overlooking real physiological effects on specific maintenance processes; and (*ii*) misinterpreting sleep–wake changes as metabolic impacts. Environmental stressors may cause metabolic effects confined to specific sleep–wake states, due to effects on specific maintenance processes, but researchers could miss these impacts if measuring metabolic rates during the wrong period. For example, aquatic acidification or salinity changes can alter ion regulation in marine organisms (Röthig *et al*., 2023), but since this may occur primarily during wakefulness, sleep‐only measurements could underestimate associated energetic costs and impacts. Conversely, some reported environmental effects on metabolism may actually reflect sleep architecture changes rather than direct physiological costs. Noise pollution, light pollution, or habitat disturbance can fragment sleep or alter time spent in different sleep states during measurement periods (Aulsebrook *et al*., 2018), leading to apparent ‘metabolic effects’ that represent shifts in sampled sleep–wake states instead of true changes in underlying maintenance costs. Climate warming might simultaneously impose real thermoregulatory costs (primarily during wakefulness) while altering sleep duration or quality, confounding direct temperature effects with sleep‐mediated measurement window changes. Indeed, environmental factors may operate through multiple pathways: direct effects on maintenance processes, indirect effects through sleep–wake architecture changes, and measurement artefacts from state‐dependent sampling (Fig. 1). Disentangling these mechanisms will be important for understanding true physiological impacts of environmental change.

## V. RE‐EVALUATING MAINTENANCE METABOLISM: A PATH FORWARD

It is worth asking: under what conditions would sleep‐state partitioning of maintenance costs not affect the measurement or interpretation of SMR? For this to be the case, several biologically implausible criteria would need to be met. First, all maintenance processes would need to operate at equivalent intensity across wakefulness, NREM, and REM sleep, or at least have equal energetic costs across these states. As we have discussed, this condition is at odds with well‐documented down‐regulation of some maintenance processes during sleep and upregulation of others. Second, individuals would need to exhibit minimal among‐ and within‐individual variation in daily sleep–wake cycles and sleep architecture (e.g. REM:NREM ratios), such that any fixed measurement window captures the same metabolic profile across animals and days. Finally, downstream uses of SMR estimates – such as comparisons across individuals or species, or calculations of aerobic scope or energy budgets – would need to be unaffected by any sleep–wake biases in SMR measurements and involve only the same behavioural or physiological states in which SMR was measured.

Taken together, these conditions are not only unlikely, but biologically unrealistic. As such, it is no longer tenable to assume that SMR reflects a fixed energetic baseline and it is critical to develop strategies that can mitigate or quantify this source of bias. Here we outline a range of possible approaches, from the ideal but logistically demanding to more feasible alternatives. The most appropriate option will also depend on factors such as cost and alignment with specific research goals.

### (1) Defining an integrated daily maintenance expenditure

Although logistically untenable in most situations, at least for now, a ‘gold standard’ for estimating maintenance metabolism would move beyond the assumption of a static maintenance cost to capture an integrated daily maintenance expenditure (IDME): the total energetic cost of maintenance processes across all sleep–wake states over a full circadian cycle. This would ideally involve continuous or high‐resolution measurement of metabolic rate across 24 h (or longer), with concurrent classification of sleep–wake state to allow state‐specific partitioning of energy use. Depending on the organism, this could be achieved using respirometry or doubly labelled water paired with electrophysiological, behavioural, or indirect indicators of sleep–wake state (Campbell & Tobler, 1984) [e.g. electroencephalography (EEG) in mammals or birds, accelerometry or infrared video tracking in fishes or invertebrates]. The goal would be not just to average metabolic rate across time, but to weight it by the proportion of time spent in each state and the specific processes active during those periods. To be clear, this is logistically challenging, or even impossible, with existing technology and especially in non‐model organisms. However, such an approach would offer the most ecologically and evolutionarily relevant estimates of baseline metabolism, reflecting how organisms actually allocate energy to maintenance functions over time, rather than how they perform in an artificially static physiological state.

A conceptual model for estimating IDME can be formalised as a time‐weighted sum of state‐specific metabolic rates:

$$\text{IDME}=\sum_{i=1}^{n}Mi\cdot Ti,$$

where M*i*  = mean metabolic rate during state *i* (e.g. wakefulness, NREM sleep, REM sleep), T*i*  = proportion of the 24‐h period spent in state *i* (such that ∑T*i* = 1), and *n* = number of behavioural states considered (typically three for mammals and birds: wake, NREM sleep, REM sleep).

This equation assumes that each behavioural state has a characteristic metabolic rate and that total maintenance cost is the sum of these rates scaled by the time spent in each state. If empirical data are available, M*i* can be measured directly; otherwise, state‐specific correction factors can be applied to standard SMR values. For example, if quiet wake SMR is used as a baseline, literature‐derived multipliers (e.g. 0.83 for NREM, 0.44 for REM, Table 1, Appendix S1) can be applied to approximate taxa and state‐specific contributions. This substitution is not ideal but is conceptually analogous to how generalised metabolic scaling exponents are often applied to data sets to correct for the effects of body mass, when data for that exact species or size range are not available. Similarly, the time allocation terms (T*i*) can be derived from electrophysiological data [e.g. EEG/electromyography (EMG) recordings in mammals], automated behavioural tracking (e.g. posture analysis or motion sensors), or estimated from published sleep‐architecture profiles for a given species. In cases where species‐specific data are unavailable, approximate values can be obtained from related taxa or scaled using known allometric or ecological correlates of sleep duration. This formulation allows estimation of daily maintenance costs in a way that reflects both temporal partitioning of behaviour and differential expression of maintenance functions across states. However, if generalised estimates for sleep‐state multipliers are being used, this would not address biases in SMR estimation that occur at the individual level, due to among‐individual variation in sleep architecture (Steinmeyer *et al*., 2012; Alós *et al*., 2017).

### (2) Enhancing current methods through sleep‐state inference

Existing approaches may be improved by developing better inferences about the sleep–wake state during SMR measurement. These refinements may serve as intermediate solutions that improve the biological realism of SMR estimates, particularly in systems where direct state identification is challenging but behavioural and metabolic data are available at high temporal resolution. In this way, existing methodologies can evolve towards more informed estimates of maintenance metabolism, even in the absence of full IDME capability.

For instance, measuring oxygen uptake across full circadian cycles may help capture a broader range of sleep–wake states (Adamovich *et al*., 2019), although without clear identification of which states are being recorded estimates will remain biased towards lower‐cost sleep phases. This is particularly relevant in intermittent‐flow respirometry, where the method of SMR calculation itself may introduce hidden state‐associated bias. Approaches such as using a lower quantile of oxygen uptake values to define SMR (Chabot, McKenzie & Craig, 2016a) may disproportionately represent sleeping periods, particularly in individuals with greater sleep needs, leading to underestimates of true time‐integrated SMR. Similarly, the use of the mean of the lowest normal distribution (Chabot *et al*., 2016a) will not resolve this issue unless data span multiple circadian cycles; even then, the resulting SMR estimate is likely to reflect metabolically quiescent phases such as REM sleep.

However, these same methods could be refined to disentangle sleep‐ and wake‐dominant energy costs. If repeated patterns emerge across the diel cycle – such as distinct frequency distribution peaks in oxygen uptake values (Adamovich *et al*., 2019), these may correspond to specific sleep–wake states and could be used to partition SMR into state‐specific components. Pairing such analyses with infrared video tracking or automated motion detection would allow coarse classification of behavioural state, helping to align metabolic estimates with sleep–wake architecture. In aquatic systems using intermittent‐flow respirometry (Svendsen, Bushnell & Steffensen, 2016; Killen *et al*., 2021), another promising strategy would be to pair activity measurements with oxygen uptake slopes on a per‐phase basis, generating a large number of slope–activity pairs which could be used to calibrate the relationship between spontaneous movement and oxygen uptake. This would allow researchers to extrapolate to an estimated SMR at zero activity, yielding a more realistic estimate of maintenance costs during wakefulness. However, this approach requires the ability to quantify and align activity rapidly with each oxygen uptake measurement. As such, it highlights the need for improved video acquisition systems and automated analytical pipelines capable of extracting activity metrics at high temporal resolution.

### (3) State‐specific metabolic profiling

While IDME offers the most comprehensive estimate of baseline energy use, it is not always necessary, or even desirable, depending on the research question. In many cases, state‐specific SMR measurements may be the most appropriate approach, particularly when the behavioural or physiological state during measurement aligns with the focal process under investigation. For example, if the aim is to understand energy constraints on locomotion, predator avoidance, or other active behaviours, SMR and aerobic scope measured during quiet wakefulness may offer more meaningful insight than a time‐averaged value diluted by metabolically depressed sleep phases. Conversely, studies focused on immunity, cellular repair, or protein synthesis may benefit from sleep‐specific SMR measurements, particularly if these processes are known to be upregulated during NREM sleep (Schmidt *et al*., 2017). Beyond their utility for improving SMR estimates, finer‐scale, state‐resolved metabolic measurements (e.g. distinguishing REM, light NREM, and deep NREM) also offer an opportunity to test new hypotheses about the energetic roles and functional significance of different sleep states. Instead of prescribing a single ideal measurement strategy, we suggest that researchers explicitly match their SMR measurement window to the behavioural or ecological state most relevant to their hypothesis, and interpret their results accordingly. This state‐matching approach offers a pragmatic and conceptually sound alternative when full 24‐h measurement is not feasible. Developing the capacity for such high‐resolution, state‐specific metabolic measurements would therefore complement IDME by providing insight into the mechanistic contributions and costs of individual sleep states.

### (4) Understanding and acknowledging the extent of bias

In some cases, simply acknowledging and quantifying these types of bias may be sufficient. Or, if researchers can confirm (or reasonably assume) that maintenance costs are similar between the measurement window and the behavioural context of interest (e.g. day *versus* night), then some level of state‐related error may be tolerable. After all, respirometry already involves accepted approximations – such as using oxygen uptake as a proxy for true energy expenditure (Svendsen *et al*., 2016). Our framework highlights an additional, but to date overlooked, source of variation that can now be assessed and, where necessary, addressed.

### (5) Avenues for future research

Confirming the extent to which SMR fluctuates with sleep–wake state across individuals, species, and contexts will require targeted empirical research (Table 2). For example, many of our estimates of state partitioning are indirect and based on up‐ or down‐regulation in organ or tissue functioning, as opposed to direct measures of state‐ and process‐dependent energy expenditure (Rolfe & Brown, 1997; Schmidt *et al*., 2017). Increased direct measurements of metabolic rate across sleep–wake states, especially using high‐resolution methods that can distinguish NREM and REM sleep, are needed to confirm the predicted shifts in energetic allocation. In addition, naturally divergent sleep architectures across species or ecotypes offer opportunities to test whether state partitioning contributes to apparent interspecific differences in SMR. For example, comparing high‐REM and low‐REM sleep phenotypes, or animals exposed to chronically fragmented *versus* consolidated sleep (Lesku *et al*., 2008; Beauchamp, 2009; Tisdale *et al*., 2018), may help disentangle true physiological divergence from measurement artefacts. Contrasting diurnal *versus* nocturnal mammals, cave *versus* surface‐dwelling morphs of the same species (e.g. Mexican cavefish, *Astyanax mexicanus*; Jaggard *et al*., 2017), or animals exposed to varying environmental conditions that alter sleep architecture (Randler, 2014; Mortlock *et al*., 2024) could also offer powerful systems for testing whether variation in REM/NREM sleep balance corresponds to predictable shifts in measured metabolic rate.

**Table 2:** Empirically testable predictions arising from a state‐partitioned view of standard metabolic rate (SMR). Implications range from experimental design to broader evolutionary and ecological theory.

Prediction	Rationale	Possible Test	Implications
SMR measured during wakefulness (e.g. extrapolating metabolic rate to zero activity) will exceed SMR measured during sleep.	Wakefulness involves higher costs for thermoregulation, sensory processing, and ion gradients.	Compare SMR from sleep *versus* quiet wake in the same individuals.	Highlights importance of behavioural state control in metabolic protocols; attempts to correlate MR with behaviour.
Endotherms will show larger errors in SMR estimation during sleep than ectotherms.	Thermoregulatory effort is downregulated during sleep in endotherms.	Compare state‐specific SMR across endotherms and ectotherms.	SMR bias may differ systematically across taxa, complicating cross‐species comparisons.
Species or individuals with higher REM sleep proportions will show greater underestimation of SMR when measured during sleep.	REM sleep is metabolically less costly; a higher proportion of REM skews measured values downward.	Measure REM proportion and compare to error magnitude.	REM duration may act as a hidden source of inter‐individual or interspecies variation.
Individuals or species with larger or more neuron‐dense brains will show a greater error in SMR estimation during sleep.	More brain ion regulation as a maintenance cost during waking.	Compare state‐specific SMR across species with different brain sizes or neuron densities.	SMR variation within and across species may be partially due to biases associated with misestimation of total brain costs.
Trait correlations with SMR (e.g. boldness, activity) depend on sleep architecture during measurement.	Sleep traits modulate the underlying maintenance processes being measured.	Control for or stratify analyses by sleep profile.	Some reported physiological–behavioural links may be artefacts of sleep‐state variation.
Apparent context‐dependent shifts in SMR–trait relationships may reflect altered sleep architecture, not true metabolic plasticity.	Environmental variables (e.g. temperature) affect sleep and thereby SMR estimates.	Simultaneously track sleep and SMR across environments.	Reframes some plasticity findings as measurement artefacts, not physiological change.
Species with high maintenance demands (e.g. immune or neural activity) will exhibit longer or more consolidated sleep.	Sleep permits efficient expression of these functions.	Correlate maintenance traits and sleep duration across species.	Suggests evolutionary linkage between sleep architecture and physiological capacity.
Sleep architecture and related SMR estimation error will show a phylogenetic signal.	Sleep–metabolism integration may follow evolutionary trajectories.	Map traits and error onto phylogenies.	Affects how metabolic traits are interpreted in a comparative or macroevolutionary context.
Environmental factors that fragment sleep will increase measured SMR.	Wake periods during measurements inflate apparent baseline metabolism.	Compare SMR and sleep in disturbed *versus* controlled settings.	Redefines ‘stress effects’ on metabolism as partly sleep modulated.
Treatment effects on metabolism will be state dependent, with sleep‐active interventions showing stronger effects during sleep measurements and wake‐active interventions during wake measurements.	Maintenance processes are temporally partitioned; interventions affecting specific processes are only detectable when those processes are most active.	Compare SMR responses to stress during sleep *versus* wake measurements.	Inconsistent treatment effects may reflect measurement timing instead of biological variation.
Animals expressing unihemispheric NREM sleep will show simultaneous state‐dependent differences in metabolic activity between hemispheres.	One hemisphere is in NREM sleep while the other remains awake, creating a built‐in comparison of sleep *versus* wake metabolic profiles within the same individual and at the same moment.	Measure hemisphere‐specific cerebral metabolic rate (e.g. *via* blood flow, temperature proxies, or neural metabolic markers) in species such as cetaceans or certain birds during unihemispheric sleep.	Provides a powerful within‐individual, real‐time test of state‐dependent maintenance costs and offers a unique system to validate predictions about sleep–wake metabolic partitioning.
Species with extremely low or extremely high daily sleep will show low within‐individual variation in SMR estimates but a consistent, state‐dependent bias.	SMR measurements repeatedly sample the same dominant physiological state (predominantly wake or predominantly sleep), reducing variance but not eliminating systematic bias towards that state's metabolic profile.	Compare repeated SMR measurements across species with extreme sleep quotas (e.g. migrating sandpipers, elephant seals, armadillos) and quantify whether variance is low but estimates are consistently shifted relative to species with balanced sleep–wake cycles.	Extreme sleepers/non‐sleepers serve as boundary cases showing that state dominance can stabilise, but still distort, SMR estimates.

Further, little is currently known about how transitions between sleep states affect maintenance costs. Our models implicitly assume a rapid or instantaneous switch in physiological function when transitioning between sleep states, but this is also unlikely to reflect biological reality. Transitional periods may involve partial or overlapping activation of maintenance processes, and in species with highly fragmented sleep, carry‐over effects between states could meaningfully alter the relative costs and timing of metabolic costs of specific processes (Randler, 2014; Schmidt *et al*., 2017). Understanding these transitional dynamics will be essential for refining both empirical measurements and modelling approaches.

From a methodological perspective, emerging tools in machine learning and miniaturised sensors will make the proposed shift toward state‐explicit metabolism both realistic and scalable. Markerless pose estimation and self‐supervised video models could be used to infer sleep–wake states (and sleep fragmentation) from behaviour alone (Nath *et al*., 2019), facilitating continuous, high‐resolution classification alongside respirometry (Rößler *et al*., 2022). Hidden Markov and switching state‐space models could then be used to align sleep–wake states with oxygen‐uptake time series to estimate state‐specific SMR (Mendez *et al*., 2010; He *et al*., 2023). Meanwhile, miniaturised sensors, such as accelerometers, heart rate loggers, ECGs, temperature loggers, and low‐power EEG/EMG for small vertebrates could be used for physiological sleep‐stage estimation in species where it was previously impossible (Yang *et al*., 2021). In fishes, for example, compact accelerometers or EMG tags can provide reliable proxies of activity and restfulness that could be aligned with oxygen‐uptake data from intermittent flow respirometry. Advancing these technologies will allow us to refine how we measure maintenance metabolism by surpassing reliance on relatively crude estimation of energy expenditure using a static SMR value, allowing the consideration of sleep–wake states during SMR estimates to become the norm rather than exceptional.

## VI. CONCLUSIONS

(1) SMR is not a fixed metabolic baseline and would be better conceptualised as dynamic and dependent on sleep–wake states. Maintenance costs are partitioned across sleep–wake states with wakefulness emphasising thermoregulation, ion and sensory processing, while sleep (especially NREM sleep) prioritises protein synthesis, cellular repair, immunity, and plasticity. As a result, how ‘maintenance metabolism’ is defined and the processes that contribute to SMR depend entirely on when the measurements are conducted and which states are active during measurements.
(2) Measuring SMR in any single state is likely to produce predictable bias. Sleep‐only SMR may underestimate integrated daily maintenance costs, while wake‐only SMR will overestimate them. The size and direction of this error are not random but track sleep architecture (e.g. REM proportion, sleep fragmentation), meaning sampling window alone can generate apparent differences among treatments, individuals, or species.
(3) Sleep architecture can confound biological comparisons and interpretation of SMR. Sleep duration, fragmentation, and REM:NREM ratios all vary with body size, age, social status, and environment, and so current SMR estimates may partly reflect sleep traits rather than intrinsic maintenance metabolism. This may help explain unexplained variance in repeatability, heritability, and body‐mass scaling of metabolic rate.
(4) The ability to detect effects of treatments or environmental factors on SMR will depend on matching measurement period to process timing. Treatments that alter wake‐active processes (e.g. thermoregulation, ion transport) will have larger effect sizes during wake, while interventions affecting sleep‐active processes (e.g. protein synthesis) are most detectable during sleep. Measuring SMR during the wrong state may cause researchers to underestimate or miss effects on SMR entirely.
(5) State‐partitioning of maintenance processes has far‐reaching consequences for interpretation of SMR across organismal biology. Because sleep architecture determines which maintenance processes are expressed during measurement, it can shift variance components, therefore reducing repeatability when sleep varies within individuals, or inflating heritability estimates when it is stable among individuals. These effects can also affect estimates of energy budgets, aerobic scope, life‐history trade‐offs, metabolic scaling, and evolutionary constraints.
(6) As a general principle, researchers should consider if and how sleep–wake state is accounted for in metabolic measurements, and the implications of state‐limited sampling on the traits and comparisons they are examining. Where possible, IDME should be measured over a full 24‐h cycle with concurrent sleep–wake state classification. When that is infeasible, report state‐matched SMR aligned to the biological context of interest, and clearly document sampling windows and any sleep indicators.
(7) By abandoning the fiction of a constant metabolic baseline, we can build a more accurate and biologically grounded understanding of organismal energetics.

## Supporting information


**Appendix S1.** Simulations of state‐dependent SMR and experimental error.
