# Supplementary material for: The myth of the metabolic baseline: sleep–wake cycles undermine a foundational assumption in organismal biology
Source: Biol Rev Camb Philos Soc. 2026 Jan 22;101(3):1491–510. doi: 10.1002/brv.70133 (PMC13149784; doi:10.1002/brv.70133)
Supplement: Supplementary file 1 — Appendix S1. Simulations of state‐dependent SMR and experimental error. [file BRV-101-1491-s001.docx]

**Appendix S1: Simulations of state-dependent SMR and experimental error**

**(1) Estimating error in SMR from state-limited sampling**

To quantify the potential error introduced when standard metabolic rate (SMR) is measured in only a single sleep–wake state, we produced a deterministic model using R (v4.4.0) to simulate how state-dependent partitioning of maintenance processes can affect SMR estimates.

We first defined 12 physiological maintenance processes contributing to SMR (e.g. ion gradient maintenance, thermoregulation, protein synthesis), based on values derived from literature estimates and physiological reasoning where required (Table 1). Each process was assigned: (1) an estimated contribution to total SMR (%); and (2) proportional activity levels across wake, non-rapid eye movement (NREM) sleep, and rapid eye movement (REM) sleep. These activity levels reflect known or inferred patterns of state-dependent up- or down-regulation (e.g. thermoregulation is active primarily during wake; protein synthesis peaks during NREM sleep).

We then simulated two contrasting SMR estimation approaches: one where measurements are taken exclusively during wakefulness (‘wake-only’), and another where measurements are taken exclusively during sleep (‘sleep-only’). In each model, metabolic costs are further partitioned between NREM and REM sleep, varying according to the proportion of total sleep time the animal spends (or would normally spend, in the case of the ‘wake-only’ panel) in REM (ranging from 10% to 50%). The true SMR for a given individual or species was modelled as the weighted sum of state-specific SMR values over a full 24-h period, representing their proportion of time spent awake *versus* asleep. Per cent error was calculated as the absolute deviation of the wake-only or sleep-only estimate from the true time-integrated SMR.

**(2) Simulating the effects of state-dependent partitioning on SMR estimation**

To explore how neglecting sleep–wake partitioning may bias estimates of SMR and aerobic scope, we constructed a stochastic individual-based simulation in R (v.4.4.0). The model generated a simulated population of 200 individuals, each undergoing five repeated measurements across five separate days (totalling 1000 observations). The true 24-h integrated SMR for each individual was drawn from a normal distribution centred at 0.35 (arbitrary units) with a standard deviation of 0.05, producing approximately 2.5-fold variation across the population.

To introduce biologically plausible within-individual consistency in sleep patterns, each individual was assigned a baseline trait value for total sleep duration and REM sleep proportion. These values were drawn from normal distributions (mean = 10 h, SD = 1 for total sleep; mean = 0.25, SD = 0.05 for REM proportion, constrained to 0.05–0.5). On each of the five simulated nights, an individual’s sleep architecture was modelled by generating nightly values from normal distributions centred around their baseline, with additional night-to-night stochasticity. Specifically, daily total sleep duration was drawn from a normal distribution centred on the individual’s baseline, with a smaller standard deviation (e.g. SD = 0.5 h), while daily REM proportion was similarly drawn from a normal distribution centred on the individual’s REM baseline (SD = 0.025), constrained between 0.05 and 0.5. This structure preserved among-individual differences in sleep architecture while allowing plausible intra-individual variation across repeated measures.

Each SMR estimate during the 12-h overnight measurement window was calculated based on the time-weighted expression of metabolic costs during REM and NREM sleep. These were assumed to reflect only partial contributions of the total maintenance processes expressed during waking hours, with multipliers derived from literature-based estimates, expressed as proportions of the maintenance costs while awake: 0.436 for REM sleep and 0.834 for NREM sleep (Table S1). These multipliers were applied to the proportion of time spent in each sleep stage during the 12-h window, relative to the individual’s true SMR. Gaussian noise (SD = 0.01) was added to all measurements to reflect routine technical error during measurements.

A single value for maximum metabolic rate (MMR) was independently generated for each individual from a log-normal distribution with a mean centred around fivefold the population mean SMR and modest variation (log SD = 0.01). For each individual on each measurement day, we calculated aerobic scope (AS) as the difference between MMR and multiple SMR estimates: the true time-integrated SMR (reflecting weighted contributions of wake, NREM, and REM states), the estimated SMR based on a simulated overnight sleep window, and three separate state-specific SMR values corresponding to wakefulness, NREM sleep, and REM sleep. This allowed us to compare how the SMR measurement state affects estimates of aerobic scope.

**(3) Estimating the impact of state-restricted SMR measurements on detection of treatment effects**

To examine how the timing of SMR measurement influences estimates of treatment effects, we developed a simulation model based on the proportional contributions of various maintenance processes to total metabolic rate during wakefulness, NREM, and REM sleep. This model was designed to assess error occurring when experimental treatments differentially affect maintenance processes that are distributed across sleep–wake states, such that single-state measurements may fail to capture the full impact on maintenance energy use. By comparing the sleep-only estimates to the integrated 24-h values, the model demonstrates how estimates of treatment effects vary not only with individual differences in sleep architecture but also as a function of the magnitude and state specificity of the treatment effect. This provides a framework for understanding how common experimental constraints can lead to systematic underestimation or misrepresentation of the metabolic consequences of a treatment.

We simulated a protein inhibitor treatment that reduced the metabolic cost of protein synthesis by 50% in all three states. Baseline state-partitioned contributions for protein synthesis were 1.8% of SMR during wake, 10.8% during NREM, and 5.4% during REM (Table 1). These values were reduced by 50% in a modified data set to represent a treatment that impairs protein synthesis across the full 24-h period. All other maintenance processes remained unchanged between control and treatment data sets. The model assumes that protein synthesis is the only directly affected process and that its proportional contributions are state dependent but additive.

To incorporate biological variability, we simulated 200 individuals, each measured on 5 separate days. On each simulated day, total sleep duration was drawn from a normal distribution with a mean of 8 h per 24 h period, and a standard deviation of 0.5 h. The proportion of sleep spent in REM was drawn from a normal distribution with a mean of 30% and standard deviation of 2.5%. These values were used to calculate state durations for wake, NREM, and REM sleep on each day. Using these proportions and the state-partitioned metabolic profiles, we calculated the individual’s total 24-h integrated daily maintenance expenditure (IDME) under both control and treatment conditions. This value reflects the sum of each state’s proportional duration multiplied by the respective state-specific maintenance costs. We then calculated the estimated treatment effects that would result if measurements were restricted to either wake only or to sleep (NREM and REM combined) only. These estimates were compared to the true integrated effect by expressing all values as a percentage of the individual’s baseline 24-h integrated SMR.
